# Effects of 14 days of prophylactic resveratrol supplementation in trained endurance runners upon the inflammatory markers TNF-a, IL1β, and IL-6 following a single bout of eccentric exercise

**DOI:** 10.1186/1550-2783-8-S1-P15

**Published:** 2011-11-07

**Authors:** Fanny Dufour, Andrew White, Stacie Urbina, Lem W Taylor, Colin D Wilborn

**Affiliations:** 1 Human Performance Laboratory, University of Mary Hardin-Baylor, Belton, TX 76513, USA; 2 Exercise Biochemistry Laboratory, University of Mary Hardin-Baylor, Belton, TX 76513, USA

## Background

Resveratrolis a natural polyphenol found in peanuts and grapes. Resveratrolpossesses antioxidative properties which have shown to reduce the oxidative damage from reactive oxygen species (ROS). Resveratrol also has the ability to attenuate inflammation via inhibiting TNF-a, IL-1β, IL-6, and blocking NF-kB activation. Resveratrol supplementation may have the potential to mitigate the damages associated with intense orprolonged exercise; consequently improving recovery. The purpose of this study is to evaluate the effects of a14 day prophylactic supplementation trans-resveratrol onTNF-a, IL-1β, and IL-6 from a single bout of eccentric exercise in traineddistance runners.

## Methods

Eight trained male distance runners ages 35 to 45 (38.13 ± 2.95yrs) were randomly assigned to consume in a double blind manner either a placebo (PL) or 1000mg of trans-resveratrol (polygonum cuspidatum)(RESV) daily for 14 days (Transmax, BiotiviaBioceuticals). Prior to supplementation participants’ height (69.5 ± 2.3in) and weight (165.2 ± 24.25lbs.) were recorded and body composition (17.75 ± 4.8BF%) was assessed using DEXA. VO_2_max (55.3 ± 6.4 ml/kg/min) was assessed using Fox and Costill protocol and 65% of VO_2_max heart rate (117±4.2 bpm) was established for use as intensity predicator in the downhill running protocol. Following 14 days of prophylactic supplementation, participants engaged in a 45 minute downhill running protocol at 65% of VO_2_max at a declined grade of 12%. Venous blood samples were taken prior to (PRE), immediately after(POST), one hour (1HR) and two hours (2HR) following the downhill protocol. Serum samples for each time point (PRE,POST, 1HR, 2HR) were assayed for TNF-a, IL-1β, and IL-6 using ELISA. Dietary analyses were conducted during the four days prior to testing to determine any antioxidant and anti-inflammatory influences within the diet.

## Results

A significant main effect for time (p = 0.003) for IL-6 (RESV: 0.613±0.253, 1.38±0.394, 1.978±0.479, 1.594±0.66; PL: 0.921±0.73, 2.25±1.05, 1.698±0.561, 1.953±1.87 pg/mL). Delta responses for IL-6 showed a 125.12% change at POST, 222.68% change at 1HR, and 160.03% at 2HR for the RESV group while the PL group showed a 144.3%, 84.36%, and 112.05% change at the same time points, respectively. No significant observationsfor time or between groups for TNF-a and IL-1β were observed. Responsefrom baseline for TNF-a showed a 10.91% change at POST, 53.33% change at 1HR, and 8.48% at 2HR for the RESV group while the PL group showed a 15.3%, -1.87%, and -8.96% change at the same time points (p > 0.05), respectively. For IL-1β, the response from baseline showed a 10.96% change at POST, 16.04% change at 1HR, and 18.18% at 2HR for the RESV group while the PL group showed a -39.67%, -31.15%, and -33.93% change at the same time points (p > 0.05), respectively.No differences were observed on pain scale values between groups resulting from the eccentric protocol (p > 0.05).

## Conclusion

The results of this study suggest that 14 days ofprophylactic Resveratrol supplementation does not attenuate inflammatory responses resulting from a single bout of eccentric exercise in trained endurance runners.

